# Global Association of Cold Spells and Adverse Health Effects: A Systematic Review and Meta-Analysis

**DOI:** 10.1289/ehp.1408104

**Published:** 2015-05-15

**Authors:** Niilo R.I. Ryti, Yuming Guo, Jouni J.K. Jaakkola

**Affiliations:** 1 Center for Environmental and Respiratory Health Research, Faculty of Medicine, University of Oulu, Oulu, Finland; 2 Medical Research Center Oulu, Oulu University Hospital and University of Oulu, Oulu, Finland; 3 Division of Epidemiology and Biostatistics, School of Public Health, University of Queensland, Brisbane, Queensland, Australia

## Abstract

**Background:**

There is substantial evidence that mortality increases in low temperatures. Less is known about the role of prolonged cold periods denoted as cold spells.

**Objective:**

We conducted the first systematic review and meta-analysis to summarize the evidence on the adverse health effects of cold spells in varying climates.

**Data sources and extraction:**

Four databases (Ovid Medline, PubMed, Scopus, Web of Science) were searched for all years and languages available. “Cold spell” was defined as an event below a temperature threshold lasting for a minimum duration of 2 days. Of 1,527 identified articles, 26 satisfied our eligibility criteria for the systematic review, and 9 were eligible for meta-analyses. The articles were grouped by the three main study questions into Overall-effect Group, Added-effect Group, and Temperature-change-effect Group.

**Data synthesis:**

Based on random-effects models in the meta-analyses, cold spells were associated with increased mortality from all or all nonaccidental causes (summary rate ratio = 1.10; 95% CI: 1.04, 1.17 based on 9 estimates from five studies), cardiovascular diseases (1.11; 95% CI: 1.03, 1.19; 12 estimates from eight studies), and respiratory diseases (1.21; 95% CI: 0.97, 1.51; 8 estimates from four studies). Estimated associations were stronger for people ≥ 65 years of age (1.06; 95% CI: 1.00, 1.12) than for people 0–64 years of age (1.01; 95% CI: 1.00, 1.03). Study-specific effect estimates from a limited number of studies suggested an increased morbidity related to cold spells, but it was not possible to quantitatively summarize the evidence.

**Conclusions:**

Cold spells are associated with increased mortality rates in populations around the world. The body of evidence suggests that cold spells also have other adverse health effects. There was substantial heterogeneity among the studies, which should be taken into account in the interpretation of the results.

**Citation:**

Ryti NR, Guo Y, Jaakkola JJ. 2016. Global association of cold spells and adverse health effects: a systematic review and meta-analysis. Environ Health Perspect 124:12–22; http://dx.doi.org/10.1289/ehp.1408104

## Introduction

There is substantial evidence from epidemiologic studies that daily mortality is related to ambient temperature during the day or preceding days (Analitis et al. 2008). The shape of the relation has been described as a reverse-J-, V-, or U-pattern, with a nonlinear increase in mortality around an optimal temperature point (Ballester et al. 2011; Curriero et al. 2002; Guo et al. 2013; Huang et al. 2012b). This shape has been observed in different climates and populations, and the optimal temperature level differs by population and climate (Ballester et al. 2011; Guo et al. 2013; Huang et al. 2012b). The differences can be relatively large even within a small geographical area: optimal temperature based on thermal comfort ranges from 14°C in central Europe to 25°C in southern Spain (Ballester et al. 2011). There is also some evidence that the adverse health effects of cold temperatures are more pronounced in warmer climates and vice versa, and thus these effects are probably not so much associated with some universal temperature level as they are with a temperature level that is relative to the prevailing climate (Ebi and Mills 2013; Eurowinter Group 1997; Guo et al. 2013). In addition to the effects of the level of temperature per se, there is some evidence that a change in temperature over time could have adverse health effects (Chen et al. 2010; Guo et al. 2011; Jaakkola et al. 2014; Tan et al. 2005; Yang et al. 2009). The effects of exposure to cold could be modified by the type of climate, season, housing conditions, or factors defining the susceptibility of the population exposed to cold spells (Conlon et al. 2011; Ebi and Mills 2013; Eurowinter Group 1997).

Physiological and pathological effects of short-term exposure to cold are well known (Holmér et al. 2012). Low atmospheric temperature induces vasoconstriction and increases systolic and diastolic blood pressure, blood viscosity, blood cholesterol, platelet count, and red blood cell count in a matter of hours (Keatinge et al. 1984; Neild et al. 1994), which may increase the risk of atherous plaque rupture, myocardial infarction, and stroke. Exposure to cool air also enhances diuresis and increases respiratory water loss, which lead to loss of extracellular water (Freund and Sawka 1996). Combined with the changed blood pressure that drives plasma fluid to the interstitial space, these changes make the blood more concentrated. For example, fibrinogen, which is a risk factor of arterial thrombosis, cannot redistribute to interstitial fluid due to its large molecule size, whereas protein C, which would inhibit several steps in the clotting process, is small enough to escape from the main circulation (Neild et al. 1994). Cold weather induces functional changes in the airways through cooling of the skin or the simultaneous cooling and drying of the nasal and airway mucosa while inhaling cold air (Eccles 2002). There is experimental evidence on responses to cold air, such as congestion and rhinorrhea in the upper airways and bronchoconstriction in the lower airways (Cruz and Togias 2008; Koskela 2007; Koskela and Tukiainen 1995). Drying of the nasal mucosa could lead to hyperosmolality, neural activation, and bronchoconstriction (Koskela 2007). These effects may trigger critical pathophysiological changes among subjects with asthma or chronic obstructive pulmonary disease, leading to exacerbations and death (Carlsen 2012; Donaldson and Wedzicha 2014). Regardless of the extensive knowledge on cold-related physiological changes, there is little understanding about which pathophysiological mechanisms are important in mediating the effects of cold weather, because there are several alternative time patterns of exposure.

Episodic events such as cold spells and heat waves seem to increase mortality in patterns that may not be explained by the traditional temperature–mortality models. This proposition originates apparently from the observation of the effects of severe heat waves in the beginning of the 21st century (Le Tertre et al. 2006; Robine et al. 2008). Whereas the effects of heat waves come within 1–2 days and are thus easier to identify, cold spells seem to be associated with mortality over a period of 2 weeks (Rocklöv and Forsberg 2008). This has made it difficult to identify the health effects and to infer causality. There has been a recent increase in the amount and quality of reports on the adverse health effects of cold spells. Some studies indicate that the quantity of excess mortality and morbidity compares with that of heat waves and may even exceed it (Huynen et al. 2001; Lin et al. 2011; Ma et al. 2011; Revich and Shaposhnikov 2010). Possibly constituting a public health threat on a major scale, these findings call forth questions of whether some of these deaths could be avoidable, and what kind of mitigation might be possible in the future.

We conducted the first, to our knowledge, systematic review and meta-analysis to summarize the evidence on the adverse health effects of cold spells in populations in varying climates. Our general study objectives were *a*) to summarize the evidence on the relation between cold spells and mortality or morbidity, *b*) to clarify the terminology and methodology used in the study topic, and *c*) to identify possible gaps in knowledge. Our specific study questions were as follows: Is there a relation between cold spells and mortality or morbidity? What is the nature, quantity, and direction of this relation? What, if any, are the modifying factors of this relation? We conducted meta-analyses of the associations between cold spells and mortality from all or all nonaccidental causes, cardiovascular diseases [*International Classification of Diseases, 10th Revision* (ICD-10) codes I00–I99], and respiratory diseases (ICD-10 J00–J99).

## Methods

This systematic review and meta-analysis is based on a review protocol accessible online (http://www.oulu.fi/cerh/node/22622). PRISMA (Preferred Reporting Items for Systematic Reviews and Meta-Analyses) and MOOSE (Meta-analysis Of Observational Studies in Epidemiology) guidelines were applied (Moher et al. 2009; Stroup et al. 2000).

*Data sources*. We conducted a systematic search of four databases [Ovid Medline (https://hsl.lib.umn.edu/biomed/help/ovid-medline), PubMed (http://www.ncbi.nlm.nih.gov/pubmed), Scopus (http://www.scopus.com), and Web of Science (http://thomsonreuters.com/en/products-services/scholarly-scientific-research/scholarly-search-and-discovery/web-of-science.html)] for all years and languages available using the search command [“cold spell*”] OR [“coldspell*”] OR [“cold wave*”] OR [“coldwave*”] OR [“cold surge*”] OR [“coldsurge*”]. To ensure that we identified all relevant articles, terms were searched from all fields, truncated, and also searched as compound words. Entries in all languages were assessed. We did not apply restrictions on the type of study or the format of the report. The search extended to February 2013. Bibliographic reference lists of all included studies were searched manually.

*Selection of articles and extraction of data*. Studies that met the following four eligibility criteria were included in our review: The study *a*) provided information on the relation between cold spells and human mortality or morbidity; *b*) was an original study that had an independent study population; *c*) had an adequate definition of cold spell, cold wave, and/or cold surge, as specified below; and *d*) applied measures of mortality or morbidity that enabled the assessment of the adverse health effects. To be included in the meta-analysis, the study also had to *e*) provide quantitative information on the relation between cold spells and mortality in an intercomparable format. Our systematic review indicated that the ratio of the mortality rates was the most suitable measure of association for the meta-analysis.

**Figure 1:**
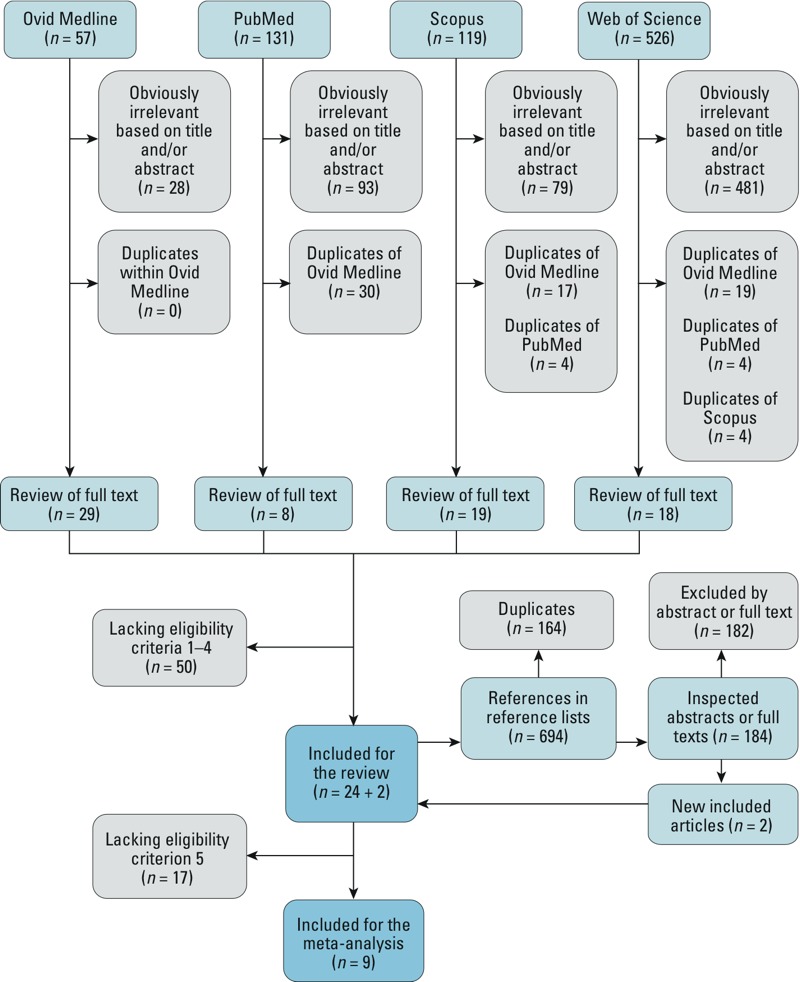
Flow diagram of study selection process. Exclusion of articles was done in three consecutive steps based on *a*) irrelevance of the title and/or the abstract, *b*) the general eligibility criteria after close inspection of the full text, and *c*) the meta-analysis eligibility criteria (meta-analysis only).

Our criteria for the adequate definition of “cold spell” constituted a combination of two components: *a*) the severity of temperature, expressed as a percentile or absolute measure; and *b*) the minimum duration of two consecutive days of the event. To acquire complete bearing of the literature, we also considered any explicit definition that was named according to our search words. Figure 1 presents a flow diagram of the study selection process.

Studies fulfilling our eligibility criteria were independently reviewed by two investigators. The main characteristics of each study were recorded to a pilot-tested form. Data were sought for quantitative information on health effects of cold spells. We applied consistent criteria for the selection of the study-specific effect estimates. First, we sought for an estimate for the total or nonaccidental mortality. Second, we identified effect estimates for the outcomes of interest, namely mortality from cardiovascular and respiratory diseases. Finally, we retrieved stratum-specific estimates for sex and age. We preferred the adjusted effect estimates over the crude estimates. Xie et al. (2013) provided city-specific effect estimates using models with different adjustments. We selected estimates that were derived by using models with uniform adjustments to all cities. Ma et al. (2012) presented results using three different reference periods. We selected estimates derived using the original reference period over estimates derived using the alternative reference periods that were used in the sensitivity analyses.

Our main principle was to use independent study-specific effect estimates—that is, to avoid the use of effect estimates representing an identical study base. Three studies presented several independent effect estimates for consideration (Huynen et al. 2001; Revich and Shaposhnikov 2008; Xie et al. 2013). Xie et al. (2013) presented city-specific effect estimates for three different cities. Revich and Shaposhnikov (2008) presented cold spell–specific effect estimates for two different cold spells. Huynen et al. (2001) presented year-specific effect estimates for cold spells occurring on different years. These observations were deemed independent and used in the meta-analyses accordingly.

*Grouping of studies and framework for meta-analysis*. In an iterative review process, we formed three groups based on the main study questions of the included articles. The Overall-effect Group assessed the overall effect of cold spells, comparing mortality rate during cold spells with mortality rate of a reference period, mainly quantifying a proportional effect (percent change in mortality rate). The Added-effect Group made an attempt to separate the possible additional effect of cold spell duration from the main effect of temperature derived from the estimated daily temperature–mortality function, as previously elaborated by Gasparrini and Armstrong (2011) for heat waves. The Temperature-change-effect Group defined cold spell as a sudden change in temperature. After formulating the three groups, we explored possibilities for quantitative synthesis within each group.

*Meta-analysis*. Although quantitative synthesis was not meaningful for all the studies in the three groups, we identified nine studies with similar study questions and comparable measures of effect. These studies, displayed in Table 1, constituted a coherent group for meta-analysis of the overall effect. The effect estimates were based on mortality rates during cold spell(s) and a reference period(s). When necessary, rate ratio estimates with 95% confidence intervals (CIs) were derived from available published data. All the studies in the meta-analyses focused on the daily number of deaths, which can be used to estimate daily mortality rates if the size of the population is assumed constant. Different approaches were used to assess the effect of cold spell on mortality, but all the effect estimates could be converted to mortality rate ratios. For example, Ma et al. (2012) directly estimated mortality rate ratios from the average daily mortality rates of cold spells and reference periods. Revich and Shaposhnikov (2008) calculated the observed numbers of death during the cold spells and estimated expected numbers from a corresponding calendar time period over 6 years. The ratio of observed and expected corresponds to mortality rate ratio.

**Table 1:** Characteristics of studies included in the systematic review and meta-analyses, Overall-effect Group.

Source	Location	Time period	Definition of cold spell (*n* of episodes)	Main outcomes and stratification by sex and age	Potential confounders taken into account	Main findings
Xie et al. 2013	3 cities in Guangdong province, China	2006–2009	Threshold: daily T_min_ < 5th percentile; duration: ≥ 5 days; (*n *= 3)	Mortality: All nonaccidental, CVD, Resp; male and female; all ages, 0–64, 65–74, ≥ 75 years	PM_10_, NO_2_, SO_2_, RH, Temporality, Influenza	Statistically significant positive association in 2 cities. Male > female; elderly > other; Resp > CVD
Ma et al. 2012	The 9 urban districts of Shanghai, China	2001–2009	Threshold: daily T_ave_ < 3rd percentile; duration: ≥ 7 consecutive days; (*n *= 1)	Mortality: All nonaccidental, CVD, stroke, CHD, Resp, COPD; male and female; all ages, 0–4, 5–44, 45–64, ≥ 65 years	Temporality	Statistically significant positive association. Male = female; elderly > other; CVD > Resp
Revich and Shaposhnikov 2010	City of Yakutsk, East Siberia, Russia	1999–2007	≥ 9 days with daily T_mean_ < 3rd percentile, of which ≥ 3 days with daily T_mean_ < 1st percentile; (*n *= 3)	Mortality: All nonaccidental, IHD, Cerebro; ages 30–64, ≥ 65 years	Temporality, Influenza	Partly statistically significant positive associations. Elderly = other; CVD > all nonaccidental; heat = cold
Chen et al. 2010	349 townships, Taiwan	1997–2003	Cold surge: fast drop in temperature (> 8°C temperature drop in 24 hr, or T_min_ < 10°C); (*n *= 13)	Mortality: CVD	Temporality	Statistically nonsignificant positive association. Social determinants had spatial nonstationary effects
Yang et al. 2009	Taiwan	2000–2003	Cold surge: fast drop in temperature (> 8°C temperature drop in 24 hr, or T_min_ < 10°C); (*n *= 4)	Mortality: CVD	Temporality	Statistically nonsignificant positive associations. A spatially varying pattern of tolerance to cold surges
Kyselý et al. 2009	Czech Republic	1986–2006	Threshold: daily T_max_ < –3.5°C; duration: ≥ 3 consecutive days; (*n *= 28)	Mortality: CVD; Male and female; All ages, 25–59, 60–69, 70–79, ≥ 80 years	Temporality	Statistically significant association in 8 of 10 population subgroups. Middle-aged men > other
Revich and Shaposhnikov 2008	City of Moscow, Russia	2000–2006	≥ 9 days with daily T_ave_ < 3rd percentile of which ≥ 6 days with daily T_ave_ < 1st percentile; (*n *= 2)	Mortality: All nonaccidental, IHD, Cerebro; ages ≥ 75 years	Temporality	Statistically significant positive association for all outcomes but only in the ≥ 75 age group
Huynen et al. 2001	The Netherlands	1979–1997	≥ 9 days with daily T_min_ ≤ –5°C, of which ≥ 6 days with daily T_min_ ≤ –10°C; (*n *= 5)	Mortality: Total, CVD, Resp, Cancer; All ages, 0–64, ≥ 65 years	Temporality	Statistically inconclusive positive association. Elderly > other; CVD > Resp
Borst et al. 1997	The Netherlands (289 nursing homes)	1993–1994	“Cold winter spells” not defined, but deductible: the coldest weeks of the study period (average of weekly T_max_ < 5°C); duration: 7 consecutive days; (*n *= not available)	Mortality: Total, CVD, Cerebro, COPD, cancer, 6 other causes; male and female; all ages, < 65, 65–74, 75–84, 85–94, ≥ 95 years	Influenza	Statistically significant positive association for total mortality and 4 out of 10 other causes. Male = female; higher age > lower age
Overall-effect Group: overall effect of a specified cold spell on mortality or morbidity compared with a reference period, either the same calendar time or a period from a comparable season. Abbreviations: Cerebro, cerebrovascular diseases; CHD, coronary heart disease; COPD, chronic obstructive pulmonary disease; CVD, cardiovascular diseases; IHD, ischemic heart disease; Influenza, the days or cases associated with influenza epidemics; NO_2_, nitrogen dioxide; PM_10_, particulate matter with aerodynamic diameter < 10 μm; Resp, respiratory diseases; RH, relative humidity; SO_2_, sulfur dioxide; T_ave_, daily average temperature; T_max_, daily maximum temperature; T_mean_, daily mean temperature; T_min_, daily minimum temperature; Temporality, long- or short-term temporal trends and/or seasonal variation and/or day of the week.						

Some studies took into account the potential induction period for the effects of cold spell by presenting the effect estimates for a “lag” period. For example, Xie et al. (2013) modeled the numbers of daily deaths in Poisson regression to estimate rate ratios for cold spell days and non–cold spell days, focusing on mortality for 27 days from the beginning of the cold spell of interest. In this case, we calculated effect estimates based on the average mortality rates (daily death counts) over the lag period. This approach provides comparable measures for the meta-analysis, although it possibly underestimates the effects by weighting all 27 days equally when the cold effect might last for a shorter period of time.

Some studies presented effect estimates only for selected groups such as men and women (Kyselý et al. 2009), certain age groups (Ma et al. 2012; Revich and Shaposhnikov 2008, 2010), or diagnoses (Borst et al. 1997; Revich and Shaposhnikov 2010). In such cases we applied a two stage meta-analysis. First, we conducted meta-analyses for all the strata with available effect estimates. Second, we summarized the data for mortality using the maximum amount of information available from the stratum-specific estimates. For example Ma et al. (2012) provided effect estimates for multiple age groups separately (0–4, 5–44, 45–64, and ≥ 65 years), without presenting an estimate for the age group 0–64 years. We used the three effect estimates (0–4, 5–44, 45–64 years) to calculate a summary-effect estimate for age group 0–64 years. Then we used the random-effects models in meta-analysis to summarize the effects of cold spells on mortality in populations of ages 0–64 and ≥ 65 years accounting for both within and between study heterogeneity. Identical approach was applied to studies presenting effect estimates for strata different from the *a priori*–specified stratification (all ages, ages 0–64 and ≥ 65 years, both sexes, males, females, cardiovascular diseases, respiratory diseases). A detailed description of the formation of the stratum specific estimates used in the two-stage meta-analyses is presented in the Supplemental Material, “Part 1” and Tables S1–S7.

Despite the two-stage meta-analyses, some of the age groups used in the meta-analyses did not fully cover the predefined strata of 0–64 and ≥ 65 years. Ages < 25 and < 30 years are missing in all effect estimates of Revich and Shaposhnikov (2010) and Kyselý et al. (2009), respectively. The effect estimate for ≥ 75 years is used to represent the age group of ≥ 65 years but not the whole age range of 0–65 years and over (Revich and Shaposhnikov 2008).

The outcomes of two studies used in the meta-analyses did not cover the whole range of diagnoses included in cardiovascular (ICD-10 I00–I99) or respiratory (J00–J99) diseases. We used the estimates for ischemic heart disease and cerebrovascular diseases (Revich and Shaposhnikov 2010), or cardiac disease and cerebrovascular accident (Borst et al. 1997) to calculate a single estimate for cardiovascular diseases for the respective studies using the two-stage meta-analysis. We also used COPD (chronic obstructive pulmonary disease) to represent respiratory diseases (Borst et al. 1997).

We conducted pre-specified stratified analyses according to the cause of mortality (ICD-10 I00–I99 cardiovascular diseases, ICD-10 J00–J99 respiratory diseases), age (0–64, ≥ 65 years), and sex. Post hoc elaboration of data revealed that meta-analyses of other groups or outcomes were not possible due to low number and heterogeneity of study-specific measures of effect.

We did not consider the effect of cold spell threshold on the summary-effect estimates because the different cold-spell groups were so diverse and included too few studies each.

We quantified heterogeneity using the Cochran Q (χ*^2^*) statistic and the *I^2^* statistic (Borenstein et al. 2009). Funnel plots for the assessment of potential publication bias were not applicable because the number of studies was small.

All analyses were conducted using R software (3.0.1) metafor package (R Core Team 2014).

## Results

*Literature search*. A step-by-step approach of the literature search is shown in Figure 1. The searches of Ovid Medline (*n* = 57), PubMed (*n* = 131), Scopus (*n* = 119), and Web of Science (*n* = 526) databases produced a total of 833 references, of which 681 were excluded based on title or abstract for being clearly irrelevant. Seventy-four studies underwent in-depth evaluation, of which 24 were deemed eligible. Two additional articles were identified through the reference lists of the included articles by using the same approach. Overall, a total of 1,527 entries were processed. Finally, 26 studies were included in the systematic review. Nine studies were included in the meta-analyses.

**Figure 2:**
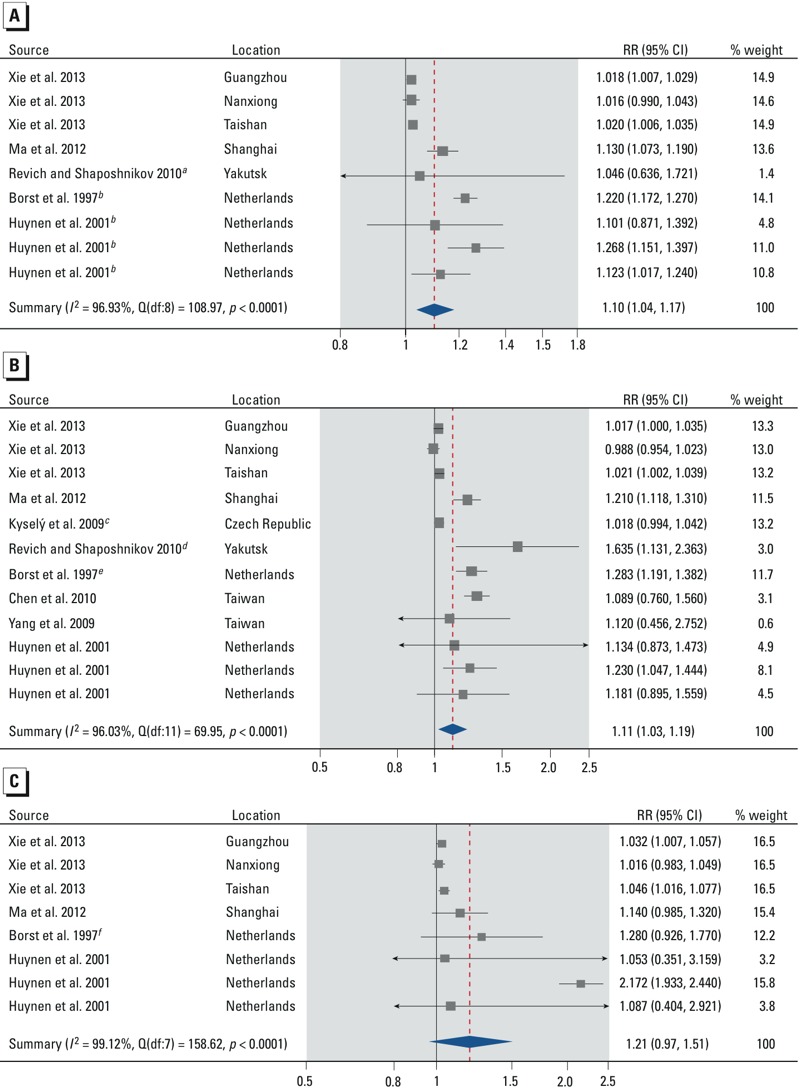
Forest plots showing the association between cold spells and mortality from all nonaccidental causes (*A*), cardiovascular diseases (*B*), and respiratory diseases (*C*) for all ages. Effect estimates are displayed on a logarithmic scale. The three independent effect estimates for Xie et al. (2013) represent cold spells in three different cities. The three independent effect estimates for Huynen et al. (2001) represent cold spells occurring in different years. Additional details about the study-specific effect estimates are provided in the Supplemental Material, “Part 1” and Tables S1–S3. Abbreviations: df, degrees of freedom; *I^2^*, total heterogeneity / total variability; Q, Q-statistic; RR, mortality rate ratio. ***^a^***The effect estimate was calculated using the effect estimates for nonaccidental mortality in the age strata 30–64 and ≥ 65 in the two-stage meta-analysis. ***^b^***The effect estimates are for mortality from all causes instead of all nonaccidental causes. ***^c^***The effect estimate was calculated using the effect estimates for nonaccidental mortality for females and males in the age stratum ≥ 25 years in the two-stage meta-analysis. ***^d^***The effect estimate was calculated using the effect estimates for ischemic heart disease and cerebrovascular diseases in the age strata 30 to ≥ 65 years in the two-stage meta-analysis. ***^e^***The effect estimate was calculated using the effect estimates for cardiac disease and cerebrovascular accident in the two-stage meta-analysis. ***^f^***The effect estimate is for mortality from COPD instead of all respiratory diseases.

*Characteristics of the studies*. There were 18 studies in the Overall-effect Group, including 9 studies estimating the overall effect of cold spells on mortality that were eligible for the meta-analyses (Table 1), and 9 that were not eligible for the meta-analyses (5 studies of mortality and 4 of morbidity, Table 2). The Added-effect Group consisted of 6 studies (Table 3), 3 of which presented effect estimates for mortality, 1 for years of life lost, and 1 for emergency department visits. The Temperature-change-effect Group consisted of 3 studies (Table 4). Two of these (Chen et al. 2010; Yang et al. 2009) defined the index period as a sudden temperature drop, but they used a concept of statistical inference and selection of reference period analogous to the Overall-effect group, as explained below in more detail. We calculated the effect estimates and respective confidence intervals from the published figures and tables of Chen et al. (2010) and Yang et al. (2009). These estimates represent the ratio of mortality rates during cold spells and the respective reference periods, and were used in the meta-analysis of cardiovascular mortality (Figure 2B; see also Supplemental Material, Table S2). One study (see Supplemental Material, Table S8) did not conform to any of the groups (Díaz et al. 2006). It fulfilled our inclusion criteria and explicitly denoted the exposure as a cold spell, but besides that, the study provided limited information.

**Table 2:** Characteristics of studies included in the systematic review but not in meta-analyses, Overall-effect Group.

Source	Location	Time period	Definition of cold spell (*n* of episodes)	Main outcomes and stratification by sex and age	Potential confounders taken into account	Main findings and effect modification
Monteiro et al. 2012	Greater Porto Metropolitan Area, Portugal	2000–2007	Comparison of several definitions; (*n *= depends on the definition)	Hospital admissions: COPD	Temporality	Moderately low T for a week > very low T lasting for a few days. Associations depend on the cold spell definition
Guo et al. 2012	City of Shanghai, China	2007–2009	Threshold: daily T_mean_ < 5th percentile; duration: ≥ 4 consecutive days; (*n *= 6)	Pediatric outpatient visits: Asthma	O_3_, RH, Temporality	Statistically significant positive association
Ma et al. 2011	City of Shanghai, China	2005–2008	Threshold: daily T_max_ and daily T_ave_ < 3rd percentile; duration: ≥ 7 consecutive days; (*n *= 1)	Hospital admissions: total, CVD, Resp	Temporality	Statistically significant positive association for all outcomes
Fitzgerald et al. 2011	State of New York, USA	1991–2006	Threshold: daily Universal Apparent Temperature UAT_mean_ < the monthly 10th percentile; duration: 3 consecutive days; (*n *= not available)	Hospitalization: asthma	H + WS included in UAT, Temporality	Statistically significant associations: positive during transitional months and negative during winter months. Both effects are larger in colder regions
Montero et al. 2010	The 5 provinces of Castile–La Mancha, Spain	1975–2003	Threshold: daily T_min_ < 5th percentile of those recorded in “winter” (= November–March); duration: not defined *a priori*; (*n *= not available)	Mortality: all nonaccidental	RH, P, Temporality, Influenza	Daily mortality increased in all provinces during cold waves
Zhong and Zhang 2009	City of Beijing, China	1998–2000	Unclear definition; (*n *= 6)	Mortality: total, all nonaccidental, CVD, CBD, AMI, Resp, COPD	Temporality	Statistically significant positive association in all outcomes presented
Plavcová and Kyselý 2009	Czech Republic and City of Prague	1992–2004	Threshold: daily T_ave_ < 5% quantile of mean annual cycle, in a given part of year; duration: ≥ 2 consecutive days; (*n *= not available)	Mortality: total, CVD, and total excluding CVD; ages 0–69, ≥ 70 years	Temporality, Influenza	No effect estimates provided. Positive association reported. Most excess mortality due to CVD. Winter > transitional months; elderly > other; country > urban
Laschewski and Jendritzky 2002	Province of Baden–Württemberg, Germany	1968–1997	Rarity-based definition: 1 cold spell in 30 years (*n *= 1); 1 cold spell every 2 years (*n *= 12); 1 cold spell every year (*n *= 31); duration: ≥ 2 consecutive days; (*n* total = 44)	Mortality: total	WVP + WS + RF included in PT, Temporality, Influenza	Point estimates of mortality rate higher during cold spell for all definitions
Institut de veille sanitaire 1988	Province of Île-de-France, France	1980–1985	“Cold wave” is not defined, but deducible. Rarity-based definition: coldest event in 30 years; duration: 16 days, not defined *a priori*; (*n *= 1)	Mortality: total, CVD, Resp, 7 subgroups of CVD, 5 subgroups of Resp, 11 other causes; male and female	Temporality	Point estimates of mortality rate higher during cold spell for 21 out of the 26 causes of death. Female > male; Resp > CVD
Overall-effect Group: overall effect of a specified cold spell on mortality or morbidity compared with a reference period, either the same calendar time or a period from a comparable season. Abbreviations: AMI, acute myocardial infarction; CBD, meaning uncertain (not specified by the authors); COPD, chronic obstructive pulmonary disease; CVD, cardiovascular diseases; H, humidity; Influenza, the days or cases associated with influenza epidemics; O_3_, ozone; P, air pressure; PT, perceived temperature; Resp, respiratory diseases; RF, radiant fluxes; RH, relative humidity; T, temperature; T_ave_, daily average temperature; T_max_, daily maximum temperature; T_mean_, daily mean temperature; T_min_, daily minimum temperature; Temporality, long- or short-term temporal trends and/or seasonal variation and/or day of the week; WS, wind speed; WVP, water vapor pressure.						

**Table 3:** Characteristics of studies included in the systematic review, Added-effect Group.

Source	Location	Time period	Definition of cold spell (*n* of episodes)	Main outcomes and stratification by sex and age	Potential confounders taken into account	Main findings and effect modification
Huang et al. 2012a	City of Brisbane, Australia	1996–2004	Threshold: daily T_mean_ ≤ 1st percentile. Also ≤ 2nd, 3rd, and 5th percentiles tried; duration: ≥ 2, ≥ 3, and ≥ 4 days; (*n *= depends on the definition)	Years of life lost: CVD	PM_10_, NO_2_, O_3_, RH, Temporality	No statistically significant associations. “Increased years of life lost are associated with cold temperatures, but there was no added effect of cold spells.”
Barnett et al. 2012	99 cities, USA	1987–2000	Threshold: daily T_ave_ < 1st to < 5th percentile; duration: ≥ 2 consecutive days; (*n *= depends on the definition)	Mortality: all nonaccidental, CVD, Resp; Ages 0–64, 65–74, ≥ 75 years	T_dew_, NO_2_, Temporality, Influenza	No statistically significant associations. “There was no increased risk of death during cold waves above the known increased risk associated with cold temperatures.”
Wang et al. 2012	4 cities, Taiwan	2000–2009	Threshold: daily T_ave_ 1st, 5th, 10th percentile; duration: ≥ 2, 2–3, ≥ 3, ≥ 4, 3–5, 6–8, ≥ 9 consecutive days; 11 combinations of the above; (*n *= not available)	Emergency department visits: all nonaccidental, CVD, Resp	PM_10_, NO_x_, O_3_, RH, WS, Temporality, Influenza	Prolonged extreme cold events were associated with increased emergency department visits. Associations depend on the cold spell definition.
Rocklöv et al. 2011	Stockholm County, Sweden	1990–2002	Threshold: daily apparent temperature < 2nd percentile; duration: 2, 3, 4, 5, 6, or 7 days; (*n *= depends on the definition)	Mortality: all nonaccidental, CVD, Resp, noncardioresp; Ages 0–44, 45–64, 65–79, ≥ 80 years	T_dew_ included in AT, NO_x_, O_3_, Temporality, Influenza	No statistically significant associations. “Extreme cold episodes contributed no additional risk compared with the risks associated with cold in general.”
Lin et al. 2011	4 cities, Taiwan	1994–2007	Threshold: T_ave_ ≤ 1st, ≤ 5th, ≤ 10th percentile; duration: 2–3 days, ≥ 4, 3–5, 6–8, ≥ 9; 8 combinations of the above; (*n *= not available)	Mortality: total, CVD, Resp	PM_10_, NO_x_, O_3_, RH, Temporality, Influenza	No statistically significant associations. “This study did not identify significant effect for stronger or prolonged cold extremes.”
Rocklöv and Forsberg 2008	Greater Stockholm, Sweden	1998–2003	Threshold: heat-wave based; duration: ≥ 2 consecutive days; (*n *= not available)	Mortality: total, CVD, Resp	Temporality, Influenza	No effect estimates provided. “No additional cold spell effect was found to be significant.”
Added-effect Group: added effect of duration of a specified cold spell on mortality or morbidity compared with the main effect of temperature on mortality, estimated from daily temperature-mortality function. Abbreviations: AT, apparent temperature; CVD, cardiovascular diseases; Influenza, the days or cases associated with influenza epidemics; NO_2_, nitrogen dioxide; NO_x_, nitrogen oxides; noncardioresp, all causes excluding cardiovascular and respiratory diseases; O_3_, ozone; PM_10_, particulate matter with aerodynamic diameter < 10 μm; Resp, respiratory diseases; RH, relative humidity; T_ave_, daily average temperature; T_dew_, dew point temperature; T_mean_, daily mean temperature; Temporality, long- or short-term temporal trends and/or seasonal variation and/or day of the week; WS, wind speed.						

**Table 4:** Characteristics of studies included in the systematic review, Temperature-change-effect Group.

Source	Location	Time period	Definition of cold spell (*n* of episodes)	Main outcomes and stratification by sex and age	Potential confounders taken into account	Main findings and effect modification
Chen et al. 2010	349 townships, Taiwan	1997–2003	Cold surge: fast drop in temperature (over 8°C temperature drop in 24 hr, or T_min_ < 10°C); (*n *= 13)	Mortality: CVD	Temporality	Statistically nonsignificant positive association. Social determinants had spatial nonstationary effects
Yang et al. 2009	Taiwan	2000–2003	Cold surge: fast drop in temperature (> 8°C temperature drop in 24 hr, or T_min_ < 10°C); (*n *= 4)	Mortality: CVD	Temporality	Statistically nonsignificant positive associations. A spatially varying pattern of tolerance to cold surges
Ha et al. 2009	City of Seoul, South Korea	1994–2006	Cold wave index (CWI) = difference between T_min_ of consecutive days; threshold derived from model fitting; (*n *= not available)	Mortality: total, CVD, Cardioresp; all ages, 0–64, ≥ 65 years	RH, Temporality	Associations between CWI and mortality. Elderly > other
Temperature-change-effect Group: overall, main, or added effect of a cold spell defined as a sudden change in temperature on mortality or morbidity. Abbreviations: Cardioresp, cardiorespiratory (cardiovascular diseases and respiratory diseases combined); CVD, cardiovascular diseases; RH, relative humidity; T_min_, daily minimum temperature; Temporality, long- or short-term temporal trends and/or seasonal variation and/or day of the week.						

The studies were conducted in 13 countries (four continents) with study periods ranging from 1968 to 2009. Effect estimates were presented for a total of 53 different health outcomes (e.g., cardiovascular mortality, cardiovascular morbidity, and emergency department visit due to cardiovascular diseases each count as one). Most studies did not follow any basic epidemiological study design such as the cohort, case–control, or case-crossover design (Maclure 1991; Rothman 2012). There were substantial differences in the cold spell definitions, reference periods, the basis of inference, and statistical methods. Comparisons between studies were difficult.

*Definition of cold spell*. There was substantial heterogeneity in the definition of a cold spell throughout the literature. The cold spells were in most studies defined statistically to constitute a set of consecutive days with extreme temperatures on the basis of a frequency distribution (e.g., 1–3 percentiles). For example, Guo et al. (2012) (Overall-effect Group) defined cold spell as ≥ 4 consecutive days with mean daily temperature below the 5th percentile of temperature during the study period 2007–2009. Chen et al. (2010) (Temperature-change-effect Group) used the cold surge definition of Central Weather Bureau in Taiwan: “a surface temperature drop within 24 hr that is greater than 8°C or the lowest temperature in the Taipei metropolitan area registering below 10°C.” All relevant definitions are presented in Tables 1–4. Additional information is provided in the Supplemental Material, Table S9.

*Definition of reference period*. There were distinctive differences in the selection of reference periods which is reflected in the basis of inference. In most cases, this information was also relatively difficult to extract from an article. The most common reference period consisted of the same calendar days of several other years (Guo et al. 2012). We denoted this concept as “annual cycle-based reference period or day,” and it represents the seasonal rarity of the event. Some studies used reference periods before and/or after the event (Chen et al. 2010; Ma et al. 2012; Yang et al. 2009). We denoted this concept “seasonally standardized reference period or days.” Other reference concepts, such as “all non-cold days of the study period” (Lin et al. 2011), or “a day or a period of lowest mortality” (Borst et al. 1997) were identified. There were also studies without a specified reference period. Instead, they fitted the temperature-mortality function and added an indicator variable for cold spell (Barnett et al. 2012).

Despite the differences in the reference periods, the studies selected for the meta-analysis shared implicitly a fundamental concept of counterfactual inference (Höfler 2005; Maclure 1998; Mittelman et al. 1993): They compared mortality during cold spells with expected mortality of the same days or periods without the cold spell. Rather than considering the heterogeneity in the type of reference period as an independent issue of validity, our emphasis was in this correspondence of the index period(s) and reference period(s) that reflect the internal validity of the study and define the exact study question(s) addressed. The reference periods and index periods of the meta-analyzed studies are presented in Supplemental Material, Table S9.

**Figure 3:**
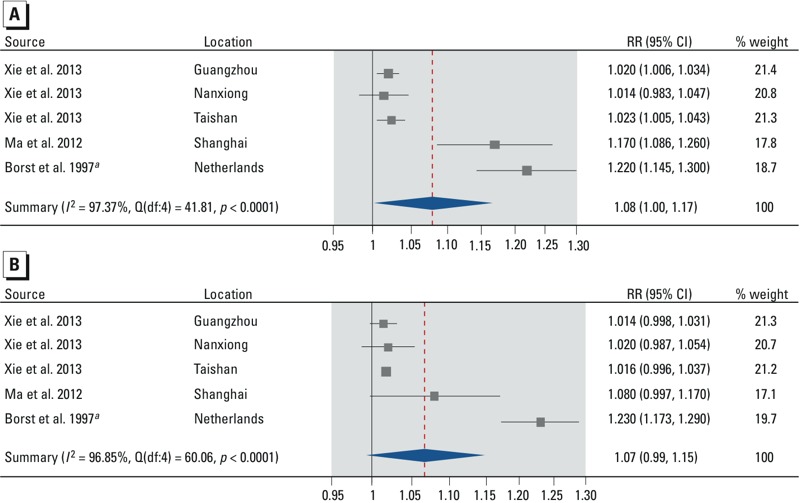
Forest plots showing the association between cold spells and mortality from all non-accidental causes by sex for males (*A*) and females (*B*), all ages. Effect estimates are displayed on a logarithmic scale. The three independent effect estimates for Xie et al. (2013) represent cold spells in three different cities. Additional details about the study-specific effect estimates are provided in the Supplemental Material, “Part 1” and Tables S4–S5. Abbreviations: df, degrees of freedom; *I^2^*, total heterogeneity / total variability; Q, Q-statistic; RR, mortality rate ratio. ***^a^***The effect estimate is for mortality from all causes instead of all nonaccidental causes.

**Figure 4:**
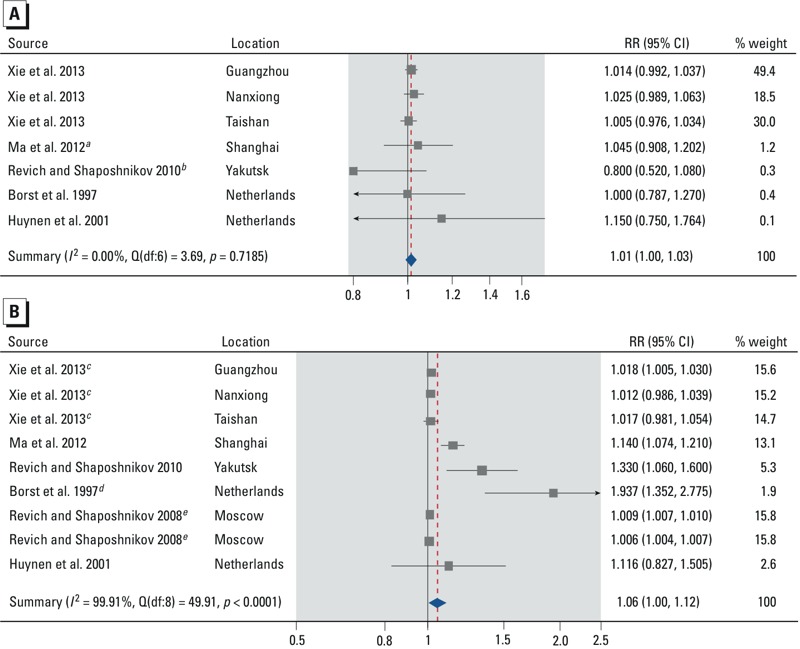
Forest plots showing the association between cold spells and mortality from all non-accidental causes by age: 0–64 years (*A*) and ≥ 65 years (*B*). Effect estimates are displayed on a logarithmic scale. The three independent effect estimates for Xie et al. (2013) represent cold spells in 3 different cities. The two independent effect estimates for Revich and Shaposhnikov (2008) represent 2 different cold spells. Additional details about the study-specific effect estimates are provided in the Supplemental Material, “Part 1” and Tables S6–S7. Abbreviations: df, degrees of freedom; *I^2^*, total heterogeneity / total variability; Q, Q-statistic; RR, mortality rate ratio. ***^a^***The effect estimate was calculated using the effect estimates for non-accidental mortality in the age strata 0–4, 5–44, and 45–64 years in the two-stage meta-analysis. ***^b^***The effect estimate is for the age stratum 30–64 years. ***^c^***The city-specific effect estimates were calculated using the city-specific effect estimates for nonaccidental mortality in the age strata 65–74 and ≥ 75 years in the two-stage meta-analysis. ***^d^***The effect estimate was calculated using the effect estimates for mortality from all causes in the age strata 65–74, 75–84, 85–94, and > 95 years in the two-stage meta-analysis. ***^e^***The cold spell-specific effect estimates are for the age stratum ≥ 75 years.

*Overall effect of cold spells*. Based on our meta-analyses, cold spells increase total and nonaccidental mortality [summary rate ratio (RR) = 1.10; 95% CI: 1.04, 1.17] (Figure 2A; see also Supplemental Material, Table S1), mortality due to cardiovascular diseases (summary RR = 1.11; 95% CI: 1.03, 1.19) (Figure 2B; see also Supplemental Material, Table S2), and mortality due to respiratory diseases (summary RR = 1.21; 95% CI: 0.97, 1.51) (Figure 2C; see also Supplemental Material, Table S3), compared with mortality rates in similar reference periods. Although all individual effect estimates but one were positive, there was substantial heterogeneity in the summary effect estimates (all heterogeneity *p*-values < 0.0001, all *I^2^* statistics > 96%). The stratified analyses show that the summary RR for total and nonaccidental mortality was slightly larger for males (summary RR = 1.08; 95% CI: 1.00, 1.17) than for females (summary RR = 1.07; 95% CI: 0.99, 1.15) (Figure 3; see also Supplemental Material, Tables S4 and S5). The summary RR was larger for people ≥ 65 years of age (summary RR = 1.06; 95% CI: 1.00, 1.12) than for people 0–64 years (summary RR = 1.01; 95% CI: 1.00, 1.03) (Figure 4; see also Supplemental Material, Tables S6 and S7). Stratification on sex and age did not reduce heterogeneity, except in the 0- to 64-year age stratum (heterogeneity *p*-value = 0.72, *I^2^* = 0%).

All of the mortality studies in the Overall-effect Group that were not included in the meta-analyses reported positive associations between cold spells and mortality (Table 2). However, effect estimates were not comparable because of heterogeneity in study designs, measures of effect, and statistical analyses.

Four studies in the Overall-effect Group investigated outcomes other than mortality, reporting positive associations between cold spells and these outcomes (Table 2). Guo et al. (2012) reported a significant relationship between cold spells and pediatric outpatient visits for asthma. Fitzgerald et al. (2011) reported a positive association between cold spells and asthma hospitalizations during the transitional months, and negative association during winter months. Monteiro et al. (2012) reported that the persistence of moderately low temperatures for a week were more significant for increasing COPD hospital admissions than very low temperatures lasting for a few days, but the observed associations depended on the cold spell definition used. Ma et al. (2011) reported that a cold spell was associated with increased risk of hospital admissions from all causes, cardiovascular diseases, and respiratory diseases.

*Added effect of cold spells*. None of the studies in the Added-effect Group found significant additional effects of prolonged cold extreme on mortality (Barnett et al. 2012; Huang et al. 2012a; Lin et al. 2011; Rocklöv and Forsberg 2008; Rocklöv et al. 2011). On the contrary, Lin et al. (2011) reported that the adverse effect on cardiovascular mortality was greater for shorter periods of extreme cold, compared with longer periods. We were unable to conduct a meta-analysis of the studies in the Added-effect Group because of the low number of comparable effect estimates. In addition, Wang et al. (2012) reported that prolonged extreme cold events were associated with increased emergency department visits.

*Main or added effect of change in temperature*. All of the three studies in the Temperature-change-effect Group reported positive associations between temperature change and the cardiovascular outcomes (Table 3). We calculated the effect estimates and confidence intervals (RR = 1.09; 95% CI: 0.76, 1.56, and RR = 1.12; 95% CI: 0.46, 2.75) from published figures and tables presented by Chen et al. (2010) and Yang et al. (2009), respectively, but no definite conclusions can be made from these two estimates with wide confidence intervals. Ha et al. (2009) reported that a temperature change is associated with increased mortality rate, but these results should be interpreted cautiously because of problems in the study design and reporting.

## Discussion

*Main findings*. Summary estimates from our systematic review and meta-analysis indicate positive associations between cold spells and mortality from all or all nonaccidental causes (RR = 1.10; 95% CI: 1.04, 1.17), cardiovascular diseases (RR = 1.11; 95% CI: 1.03, 1.19), and respiratory diseases (RR = 1.21; 95% CI: 0.97, 1.51), although the summary effect estimates showed substantial heterogeneity (all *p* < 0.0001). The studies were conducted in varying climates and diverse populations and social and environmental conditions, which may explain the heterogeneity of the effect estimates from individual studies. However, almost all effect estimates indicated an increased risk, thus conveying the similar message of adverse effects. Because we observed stronger associations with deaths due to cardiovascular and respiratory diseases than with deaths due to all or all nonaccidental causes, we assume that individuals with cardiovascular or respiratory disease are more susceptible to the adverse effects of cold spells than are healthy individuals. The summary RR for total and nonaccidental mortality was slightly larger for males (summary RR = 1.08; 95% CI: 1.00, 1.17) than for females (summary RR = 1.07; 95% CI: 0.99, 1.15), but this difference should not be deemed significant given the heterogeneity involved. The summary RR was also larger for people ≥ 65 years of age (RR = 1.06; 95% CI: 1.00, 1.12) than for people 0–64 years (RR = 1.01; 95% CI: 1.00, 1.03) (Figure 4; see also Supplemental Material, Tables S6 and S7). This finding is more convincing, although the estimate for the older age group is much less precise than the estimate for the younger group. Stratification on sex and age did not reduce heterogeneity, except in the 0- to 64-year age stratum (heterogeneity *p*-value = 0.72, *I^2^* = 0%). There is also suggestive evidence from individual studies that cold spells increase mortality and morbidity from diseases other than those used in the quantitative syntheses (Tables 1–4), but the quantification of these effects was not possible due to low number of comparable effect estimates. The added effect of duration (Added-effect Group) was reported to be null to minimal in all studies except for the one studying emergency department visits (Wang et al. 2012). However, these studies do not provide effect estimates which would allow assessment of the error marginal of their “null” findings or meta-analysis, and in our opinion these results on duration should not be considered conclusive. The three available studies in the Temperature-change-effect Group suggest that a sudden change in temperature might cause an increase in mortality. A tempting synthesis of these findings would be that extended periods of cold temperature increase mortality, and that this can be mostly explained by the cold temperature itself. However, as we will point out in the synthesis, neither of these conclusions can be made as such.

*Validity of results*. The strengths of our systematic review include selection of studies based on an exhaustive and clearly defined search strategy, including use of four main databases and cited articles from publications identified in the primary search. Two reviewers checked independently the eligibility of each article and identified the study-specific effect estimates to be used in the meta-analysis. We followed the MOOSE (Stroup et al. 2000) and PRISMA (Moher et al. 2009) guidelines.

The major limitations of our systematic review were due to the heterogeneity of cold spell definitions, reference periods, and consequently the basis of inference in the specific studies as well as limitations of the availability of suitable effect estimates for quantitative analyses. Consequently it was not possible to assess study quality using the Newcastle–Ottawa Scale or any other quality scale. We addressed the heterogeneity between studies by defining specific study questions amenable to each type of study, which resulted in three groups. We could not formally assess the role of publication bias because of the limited number of studies available.

*Synthesis with previous knowledge*. Our meta-analysis was based on studies that compared differences in mortality rates during the cold spells and the reference periods. The observed differences have usually been interpreted as effects of low temperature (Xie et al. 2013). However, the results do not provide direct information on the effect of absolute temperature per se because of three reasons: *a*) The effect estimates in the specific studies are not calculated for differences in mortality per degree in temperature; *b*) the use of duration in the cold spell definition enables possibilities that the temperature of singular days during reference periods can be as cold as or colder than the temperature of singular days during the cold spell; and *c*) the contrasts in temperature between cold spells and reference periods vary substantially in the quantity and range of temperature, as a result of certain approaches in choosing the cold spell and reference periods.

Six studies reported on the role of duration of cold spell as an additional effect. None of these provided evidence that duration would increase mortality more than expected from the additive effect of individual days. However, even in the absence of added effect in the Added-effect Group, the results should not be extended to deduce that the findings in the Overall-effect Group can be explained as the effects of daily temperature. In the Added-effect Group, the studies present the overall effect (E_O_) as the sum of the main effect of the level of daily temperature (E_T_) and the additional effect of the duration (E_D_) of the cold spell:

E_O_ = E_T_ + E_D_. [1]

In the absence of added effect (E_D_ = 0),

E_O_ = E_T_. [2]

This has often been inferred to indicate that the overall effect of cold spells is explained by the effects of average temperature only. However, the presence of any third component explaining the overall effect of cold spells—for example, a change in temperature (E_C_)—will question the correctness of the inference:

E_O_ = E_T_ + E_D_ + E_C_ [3]

⇔ E_O_ – E_D_ ≠ E_T_. [4]

For this reason, a direct comparison of the Overall-effect Group and the Added-effect Group is not sufficient to resolve the issue of whether other factors besides daily temperature explain the overall effect observed in the studies in the Overall-effect Group.

In addition to the level of temperature and duration, other characteristics of cold spells may also contribute to the adverse effects. A fast drop in temperature could be an important factor for both physiological reasons and reasons related to adaptation to environmental changes. The three studies in the Temperature-change-effect Group indicate that a change in temperature could play a role in the adverse effects. Also, there is always a temperature change of some magnitude and duration leading to the threshold temperature level that is inbuilt in the cold spell definition, regardless of whether this change is captured by the study design. However, adding another component such as a potentially harmful pattern of weather (E_P_) to Equation 3, and following the previous deduction, we can reason that it cannot be retrospectively assessed whether some of the overall effect observed in the Overall-effect Group might be explained by a change in temperature.

We suggest that the observed differences in mortality rates between the cold spells and the reference periods constitute an overall effect that can be divided into the following components: *a*) main effect of daily temperature level, *b*) possible added effect of prolonged duration of temperature level, *c*) possible main or added effects of change in temperature, and *d*) possible patterns of the three components. All these components could independently, or as modifiers, trigger the pathological pathways leading to the adverse health effects observed during or after cold spells.

In addition, some factors may modify the effects of cold spells, such as the type of climate, season, housing conditions, or the susceptibility of the population. It is also unclear whether the adverse effects are triggered by indoor or outdoor exposures.

*Questions for prevention and future research: time, place, and persons*. There is consistent evidence that cold weather increases mortality and morbidity, especially from cardiovascular and respiratory diseases. Several countries in Europe and North America have already implemented prevention programs to reduce adverse effects of cold weather (Conlon et al. 2011; Laaidi et al. 2013). The short-term measures are directed at socially deprived or homeless people, and the general population is informed about protection against cold through public national broadcast. The main long-term measures include improvements in housing insulation and heating (Laaidi et al. 2013). Important questions for prevention are, on one hand, to what extent cold spells with serious health effects can be predicted, and, on the other hand, how to protect public from the adverse health effects.

Weather stations that measure all relevant temperature and weather variables on a daily level already exist in most parts of the world (Peel et al. 2007). For prediction and evidence-based warning and action, we need to be able to characterize the phenomenon, preferably to know the pathophysiological basis of the effects and have feasible short and long-term measures to protect the public.

In our systematic review and meta-analysis we have identified fundamental gaps in the knowledge of the health effects of cold spells that impede effective prevention and therefore call for further research. Applying the principles of classical epidemiologic thinking (MacMahon and Pugh 1970), we present the open questions as descriptive of time, place, and persons:

Time: What are the harmful time patterns of cold weather? Our meta-analysis provides evidence of an overall effect of cold spell on mortality, but the relevant patterns of exposure and induction periods remain unclear. For example, we do not know the role of the duration of personal exposure to cold and how it is related to the duration of cold spells defined statistically. It is likely that the induction period—that is, the duration of cold exposure needed for causing an adverse health effect—varies substantially according to the type of health outcome. In addition, changes in exposure could be relevant as suggested by the review. Further studies need to address the role of cold exposure patterns over several days in the increase of cold weather–related mortality. These studies should take into account that each exposure–outcome relation has a specific induction period.

Place: What are the harmful places during the cold-related weather episodes? Although an increase in mortality coincides with extended periods of cold weather, there is no information on the relevant sites of exposure. One could hypothesize that time spent indoors increases during cold weather and leads to hazardous indoor exposures, such as combustion products from additional heating. Increased time in confined spaces may also increase exposure to harmful pathogenic viruses. An alternative hypothesis could be that sudden changes in temperature taking place when going out or entering indoors during cold weather are important determinants of mortality. Further studies need to elaborate the role of indoor and outdoor exposures and the risk of death during cold weather.

Persons: How should different people be protected? Although the reviewed studies did not statistically assess individual susceptibility (interaction), we assume on the basis of the observed main effects that individuals with a cardiovascular or respiratory disease are more susceptible to the adverse effects of cold spells than are healthy individuals. To focus the preventive actions on susceptible individuals and to provide useful advice, we need to better understand the underlying pathophysiological mechanisms for the effects of extended cold weather as well as characteristics of individual susceptibility. There is extensive knowledge about the effects of short-term cold exposure on cardiovascular and respiratory systems, but less is known about the most relevant responses related to cold weather because there are several alternative time patterns and places of exposure. Further studies are needed to assess the physiological responses to different characteristics of cold weather among the healthy and individuals with chronic cardiovascular and respiratory diseases.

Any preventive action directed at populations, susceptible groups, or individuals needs to be critically evaluated before general implementation.

## Conclusions

Our systematic review and meta-analysis provided evidence that cold spells are associated with increased mortality rates in populations around the world. People with cardiovascular and respiratory diseases and the elderly are potentially more susceptible to the effects of cold spells. The current body of evidence does not satisfactorily resolve the question of whether the duration of cold spells results in an added effect beyond the effect predicted from the temperature–mortality functions. There is suggestive evidence that a change in temperature might contribute to the effects of cold spells on mortality. Better understanding of the entire weather phenomenon is needed to develop the existing and new early warning systems and to give evidence-based advice on how to protect from the effects of cold spells.

## Supplemental Material

(427 KB) PDF
