# Supplementary material for: Global Association of Cold Spells and Adverse Health Effects: A Systematic Review and Meta-Analysis
Source: Environ Health Perspect. 2015 May 15;124(1):12–22. doi: 10.1289/ehp.1408104 (PMC4710591; doi:10.1289/ehp.1408104)
Supplement: (427 KB) PDF [file ehp.1408104.s001.acco.pdf]

Environ Health Perspect
doi: 10.1289/ehp.1408104

Note to Readers: *EHP* strives to ensure that all journal content is accessible to all readers. However, some figures and Supplemental Material published in *EHP* articles may not conform to 508 standards due to the complexity of the information being presented. If you need assistance accessing journal content, please contact ehp508@niehs.nih.gov. Our staff will work with you to assess and meet your accessibility needs within 3 working days.

Supplemental Material

Global Association of Cold Spells and Adverse Health Effects: A Systematic Review and Meta-Analysis

Niilo R.I. Ryti, Yuming Guo, and Jouni J.K. Jaakkola

Table of Contents

Part 1. Formation of the stratum-specific effect estimates in the two-stage meta-analysis.

Table S1. Primary values derived from the original articles and effect estimates used in the meta-analysis: all non-accidental causes.

Table S2. Primary values derived from the original articles and effect estimates used in the meta-analysis: cardiovascular diseases.

Table S3. Primary values derived from the original articles and effect estimates used in the meta-analysis: respiratory diseases.

Table S4. Primary values derived from the original articles and effect estimates used in the meta-analysis: males, all non-accidental causes.

Table S5. Primary values derived from the original articles and effect estimates used in the meta-analysis: females, all non-accidental causes.

Table S6. Primary values derived from the original articles and effect estimates used in the meta-analysis: ages 0-64, all non-accidental causes.

Table S7. Primary values derived from the original articles and effect estimates used in the meta-analysis: ages 65+, all non-accidental causes.

Table S8. Characteristics of studies included in the systematic review but not in a group, complementary information.

Table S9. Estimation of mortality in the index periods (cold spells) and reference periods for the studies in the meta-analyses.

References

Part 1. Formation of the stratum-specific effect estimates in the two-stage meta-analysis.

Xie et al. (2013) provided effect estimates in the age strata 0-64, 65-74, and ≥75 without presenting an estimate for ages ≥65. We used the effect estimates for the age groups 65-74 and ≥75 to calculate a summary-effect estimate for the age group ≥65 using the two-stage meta-analysis (Table S7). The three effect estimates for Xie at al. (2013) present in figures (2-4), and tables (S1-S6) represent cold spells in different cities.

Ma et al. (2012) provided effect estimates for multiple age groups (0-4, 5-44, 45-64, and ≥65) without presenting an estimate for the age group 0-64. We used the effect estimates for the age groups 0-4, 5-44, and 45-64 to calculate a summary-effect estimate for age group 0-64 using the two-stage meta-analysis (Table S6).

Revich and Shaposhnikov (2010) provided effect estimates in two age strata, 30-64 and ≥65. We used the effect estimates for the age groups 30-64 and ≥65 to calculate a summary-effect estimate for the whole age range using the two-stage meta-analysis (Tables S1 and S2). The original estimate for ages 30-64 was deemed representative of ages 0-64 and used in the meta-analyses as such (Table S6). It should be noted that Revich and Shaposhnikov (2010) reported that the number of deaths under 30 years was too small to analyze. Effect estimates were also provided for diagnoses that did not cover the whole range of cardiovascular (ICD-10 I00-I99) diseases. We used the effect estimates for diagnoses of ischemic heart disease and cerebrovascular diseases to calculate a summary-effect estimate for cardiovascular diseases using the two-stage meta-analysis (Table S2). Revich and Shaposhnikov (2010) reported that based on absolute numbers of deaths, these two were the most significant causes among cardiovascular deaths.

Kyselý et al. (2009) provided effect estimates for cardiovascular mortality stratified by sex, without presenting an estimate for both sexes combined. We used the effect estimates for males (all ages) and females (all ages) to calculate a summary-effect estimate for cardiovascular mortality using the two-stage meta-analysis (Table S2). It should be noted that Kyselý et al. (2009) did not include age groups 0-24 in their analyses because of the very small sample size of this group in their cardiovascular mortality data.

Revich and Shaposhnikov (2008) provided effect estimates only in the age stratum of ≥75 years. The effect estimates in the age stratum ≥75 were deemed representative of the age stratum ≥65 years but not of the whole age range 0 to 65 and over, and were used in the meta-analyses accordingly. The two effect estimates for Revich and Shaposhnikov (2008) present in figures and tables represent 2 different cold spells (Table S7).

Huynen et al. (2001) provided effect estimates for independent cold spells occurring on different years (Tables S1-S3). These observations were deemed independent and used in the meta-analyses accordingly. Suitable age-stratified effect estimates were available for one year only (Tables S6 and S7).

Borst et al. (1997) provided effect estimates for age strata <65, 65-74, 75-84, 85-94, and >95 without presenting an effect estimate for ages ≥65. We used the effect estimates for the age groups 65-74, 75-84, 85-94, and >95 to calculate a summary-effect estimate for age group ≥65 using the two-stage meta-analysis (Table SS7). Borst et al. (1997) provided effect estimates for diagnoses that did not cover the whole range of cardiovascular (ICD-10 I00-I99) or respiratory (J00-J99) diseases. We used the effect estimates of cardiac disease and cerebrovascular accident

(Table S2) to calculate a summary-effect estimate for cardiovascular diseases using the two stage meta-analysis. We used COPD to represent respiratory diseases (Table S3).

Table S1. Primary values derived from the original articles and effect estimates used in the meta-analysis: all non-accidental causes.

Source	Location	Cause of Death	Gender	Age	Rate Ratio (95% CI)	Effect Type	Lag Days	Secondary-Rate Ratio (95% CI)
Xie et al. 2013	Guangzhou	Non-accidental	both	all	1.60 (1.19, 2.14)	cumulative	27	1.018 (1.007, 1.029)
Xie et al. 2013	Nanxiong	Non-accidental	both	all	1.55 (0.77, 3.11)	cumulative	27	1.016 (0.990, 1.043)
Xie et al. 2013	Taishan	Non-accidental	both	all	1.72 (1.17, 2.55)	cumulative	27	1.020 (1.006, 1.035)
Ma et al. 2012	Shanghai	Non-accidental	both	all	1.13 (1.07, 1.19)	daily	0	1.130 (1.073, 1.190)
Revich and Shaposhnikov 2010	East Siberia	Non-accidental	both	30–64	0.80 (0.52, 1.08)	daily	0	↴
Revich and Shaposhnikov 2010	East Siberia	Non-accidental	both	65+	1.33 (1.06, 1.60)	daily	0	1.046 (0.636, 1.721)
Borst et al. 1997	Netherlands	All	both	all	1.22 (1.17, 1.27)	daily	0	1.220 (1.172, 1.270)
Huynen et al. 2001	Netherlands	All	both	all	1.101 (0.942, 1.392)	daily	0	1.101 (0.871, 1.392)
Huynen et al. 2001	Netherlands	All	both	all	1.268 (1.063, 1.397)	daily	0	1.268 (1.151, 1.397)
Huynen et al. 2001	Netherlands	All	both	all	1.123 (0.769, 1.240)	daily	0	1.123 (1.017, 1.240)

Abbreviations: Rate Ratio, mortality-rate ratio, derived from the original article; Secondary-Rate Ratio, study-specific effect estimate used in the meta-analysis; 95% CI, 95% confidence interval; "↴" indicates that more than one value contributed to the study-specific effect estimate presented below the arrow. Definitions: Effect Type = cumulative if the effect estimate presented in the study was calculated for a time period; Effect Type = daily if the effect was estimated per one day; Lag Days, the number of days used in the conversion of a cumulative measure to a daily measure.

Table S2. Primary values derived from the original articles and effect estimates used in the meta-analysis: cardiovascular diseases.

Source	Location	Cause of Death	Gender	Age	Rate Ratio (95% CI)	Effect Type	Lag Days	Summary-Rate Ratio (95% CI)
Xie et al. 2013	Guangzhou	CVD	both	all	1.59 (0.99, 2.55)	cumulative	27	1.017 (1.000, 1.035)
Xie et al. 2013	Nanxiong	CVD	both	all	0.72 (0.28, 1.85)	cumulative	27	0.988 (0.954, 1.023)
Xie et al. 2013	Taishan	CVD	both	all	1.73 (1.06, 2.83)	cumulative	27	1.021 (1.002, 1.039)
Ma et al. 2012	Shanghai	CVD	both	all	1.21 (1.12, 1.31)	daily	0	1.210 (1.118, 1.310)
Kysely et al. 2009	Czech Republic	CVD	male	all	1.063 (1.042, 1.083)	cumulative	2	↴
Kysely et al. 2009	Czech Republic	CVD	female	all	1.063 (1.044, 1.082)	cumulative	9	1.018 (0.994, 1.042)
Revich and Shaposhnikov 2010	East Siberia	IHD	both	30–64	1.78 (1.19, 2.37)	daily	0	↴
Revich and Shaposhnikov 2010	East Siberia	IHD	both	65+	1.60 (1.05, 2.15)	daily	0	↴
Revich and Shaposhnikov 2010	East Siberia	Cerebro	both	30–64	0.65 (0.00, 1.45)	daily	0	↴
Revich and Shaposhnikov 2010	East Siberia	Cerebro	both	65+	2.31 (1.63, 2.99)	daily	0	1.635 (1.131, 2.363)
Borst et al. 1997	Netherlands	CD	both	all	1.31 (1.18, 1.44)	daily	0	↴
Borst et al. 1997	Netherlands	Cerebro	both	all	1.24 (1.10, 1.40)	daily	0	1.283 (1.191, 1.382)
Chen et al. 2010	Taiwan	CVD	both	all	1.089 (0.760, 1.560)	daily	0	1.089 (0.760, 1.560)
Yang et al. 2009	Taiwan	CVD	both	all	1.120 (0.456, 2.752)	daily	0	1.120 (0.456, 2.752)
Huynen et al. 2001	Netherlands	CVD	both	all	1.134 (0.943, 1.473)	daily	0	1.134 (0.873, 1.473)
Huynen et al. 2001	Netherlands	CVD	both	all	1.230 (1.020, 1.444)	daily	0	1.230 (1.047, 1.444)
Huynen et al. 2001	Netherlands	CVD	both	all	1.181 (0.953, 1.559)	daily	0	1.181 (0.895, 1.559)

Abbreviations: Rate Ratio, mortality-rate ratio, derived from the original article; Secondary-Rate Ratio, study-specific effect estimate used in the meta-analysis; 95% CI, 95% confidence interval; "↴" indicates that more than one value contributed to the study-specific effect estimate presented below the arrow; CVD, cardiovascular diseases; IHD, ischemic heart disease; Cerebro, cerebrovascular disease or cerebrovascular accident; CD, cardiac disease. Definitions: Effect Type = cumulative if the effect estimate presented in the study was calculated for a time period; Effect Type = daily if the effect was estimated per one day; Lag Days, the number of days used in the conversion of a cumulative measure to a daily measure.

Table S3. Primary values derived from the original articles and effect estimates used in the meta-analysis: respiratory diseases.

Source	Location	Cause of Death	Gender	Age	Rate Ratio (95% CI)	Effect Type	Lag Days	Secondary-Rate Ratio (95% CI)
Xie et al. 2013	Guangzhou	Resp	both	all	2.33 (1.22, 4.46)	cumulative	27	1.032 (1.007, 1.057)
Xie et al. 2013	Nanxiong	Resp	both	all	1.53 (0.63, 3.68)	cumulative	27	1.016 (0.983, 1.049)
Xie et al. 2013	Taishan	Resp	both	all	3.38 (1.54, 7.41)	cumulative	27	1.046 (1.016, 1.077)
Ma et al. 2012	Shanghai	Resp	both	all	1.14 (0.98, 1.32)	daily	0	1.140 (0.985, 1.320)
Borst et al. 1997	Netherlands	COPD	both	all	1.28 (0.92, 1.77)	daily	0	1.280 (0.926, 1.770)
Huynen et al. 2001	Netherlands	Resp	both	all	1.053 (0.635, 3.159)	daily	0	1.053 (0.351, 3.159)
Huynen et al. 2001	Netherlands	Resp	both	all	2.172 (1.332, 2.440)	daily	0	2.172 (1.933, 2.440)
Huynen et al. 2001	Netherlands	Resp	both	all	1.087 (0.675, 2.921)	daily	0	1.087 (0.404, 2.921)

Abbreviations: Rate Ratio, mortality-rate ratio, derived from the original article; Secondary-Rate Ratio, study-specific effect estimate used in the meta-analysis; 95% CI, 95% confidence interval; "↴" indicates that more than one value contributed to the study-specific effect estimate presented below the arrow; Resp, respiratory diseases; COPD, chronic obstructive pulmonary disease. Definitions: Effect Type = cumulative if the effect estimate presented in the study was calculated for a time period; Effect Type = daily if the effect was estimated per one day; Lag Days, the number of days used in the conversion of a cumulative measure to a daily measure.

Table S4. Primary values derived from the original articles and effect estimates used in the meta-analysis: males, all non-accidental causes.

Source	Location	Cause of Death	Gender	Age	Rate Ratio (95% CI)	Effect Type	Lag Days	Secondary-Rate Ratio (95% CI)
Xie et al. 2013	Guangzhou	Non-accidental	male	all	1.70 (1.17, 2.48)	cumulative	27	1.020 (1.006, 1.034)
Xie et al. 2013	Nanxiong	Non-accidental	male	all	1.46 (0.62, 3.43)	cumulative	27	1.014 (0.983, 1.047)
Xie et al. 2013	Taishan	Non-accidental	male	all	1.87 (1.13, 3.09)	cumulative	27	1.023 (1.005, 1.043)
Ma et al. 2012	Shanghai	Non-accidental	male	all	1.17 (1.09, 1.26)	daily	0	1.170 (1.086, 1.260)
Borst et al. 1997	Netherlands	All	male	all	1.22 (1.14, 1.30)	daily	0	1.220 (1.145, 1.300)

Abbreviations: Rate Ratio, mortality-rate ratio, derived from the original article; Secondary-Rate Ratio, study-specific effect estimate used in the meta-analysis; 95% CI, 95% confidence interval; "↴" indicates that more than one value contributed to the study-specific effect estimate presented below the arrow. Definitions: Effect Type = cumulative if the effect estimate presented in the study was calculated for a time period; Effect Type = daily if the effect was estimated per one day; Lag Days, the number of days used in the conversion of a cumulative measure to a daily measure.

Table S5. Primary values derived from the original articles and effect estimates used in the meta-analysis: females, all non-accidental causes.

Source	Location	Cause of Death	Gender	Age	Rate Ratio (95% CI)	Effect Type	Lag Days	Secondary-Rate Ratio (95% CI)
Xie et al. 2013	Guangzhou	Non-accidental	female	all	1.47 (0.96, 2.26)	cumulative	27	1.014 (0.998, 1.031)
Xie et al. 2013	Nanxiong	Non-accidental	female	all	1.71 (0.71, 4.11)	cumulative	27	1.020 (0.987, 1.054)
Xie et al. 2013	Taishan	Non-accidental	female	all	1.55 (0.89, 2.70)	cumulative	27	1.016 (0.996, 1.037)
Ma et al. 2012	Shanghai	Non-accidental	female	all	1.08 (1.00, 1.17)	daily	0	1.080 (0.997, 1.170)
Borst et al. 1997	Netherlands	All	female	all	1.23 (1.17, 1.29)	daily	0	1.230 (1.173, 1.290)

Abbreviations: Rate Ratio, mortality-rate ratio, derived from the original article; Secondary-Rate Ratio, study-specific effect estimate used in the meta-analysis; 95% CI, 95% confidence interval; "↴" indicates that more than one value contributed to the study-specific effect estimate presented below the arrow. Definitions: Effect Type = cumulative if the effect estimate presented in the study was calculated for a time period; Effect Type = daily if the effect was estimated per one day; Lag Days, the number of days used in the conversion of a cumulative measure to a daily measure.

Table S6. Primary values derived from the original articles and effect estimates used in the meta-analysis: ages 0-64, all non-accidental causes.

Source	Location	Cause of Death	Gender	Age	Rate Ratio (95% CI)	Effect Type	Lag Days	Secondary-Rate Ratio (95% CI)
Xie et al. 2013	Guangzhou	Non-accidental	both	0–64	1.47 (0.80, 2.68)	cumulative	27	1.014 (0.992, 1.037)
Xie et al. 2013	Nanxiong	Non-accidental	both	0–64	1.97 (0.74, 5.25)	cumulative	27	1.025 (0.989, 1.063)
Xie et al. 2013	Taishan	Non-accidental	both	0–64	1.13 (0.53, 2.44)	cumulative	27	1.005 (0.976, 1.034)
Ma et al. 2012	Shanghai	Non-accidental	both	0–4	1.33 (0.50, 3.55)	daily	0	↴
Ma et al. 2012	Shanghai	Non-accidental	both	5–44	0.85 (0.53, 1.37)	daily	0	↴
Ma et al. 2012	Shanghai	Non-accidental	both	45–64	1.06 (0.91, 1.23)	daily	0	1.045 (0.908, 1.202)
Revich and Shaposhnikov 2010	East Siberia	Non-accidental	both	30–64	0.80 (0.52, 1.08)	daily	0	0.800 (0.520, 1.080)
Borst et al. 1997	Netherlands	All	both	<65	1.00 (0.80, 1.27)	daily	0	1.000 (0.787, 1.270)
Huynen et al. 2001	Netherlands	All	both	0–64	1.150 (0.882, 1.764)	daily	0	1.150 (0.750, 1.764)

Abbreviations: Rate Ratio, mortality-rate ratio, derived from the original article; Secondary-Rate Ratio, study-specific effect estimate used in the meta-analysis; 95% CI, 95% confidence interval; "↴" indicates that more than one value contributed to the study-specific effect estimate presented below the arrow. Definitions: Effect Type = cumulative if the effect estimate presented in the study was calculated for a time period; Effect Type = daily if the effect was estimated per one day; Lag Days, the number of days used in the conversion of a cumulative measure to a daily measure.

Table S7. Primary values derived from the original articles and effect estimates used in the meta-analysis: ages 65+, all non-accidental causes.

Source	Location	Cause of Death	Gender	Age	Rate Ratio (95% CI)	Effect Type	Lag Days	Secondary-Rate Ratio (95% CI)
Xie et al. 2013	Guangzhou	Non-accidental	both	65–74	1.85 (0.97, 3.51)	cumulative	27	↴
Xie et al. 2013	Guangzhou	Non-accidental	both	75+	1.53 (1.05, 2.24)	cumulative	27	1.018 (1.005, 1.030)
Xie et al. 2013	Nanxiong	Non-accidental	both	65–74	1.82 (0.65, 5.09)	cumulative	27	↴
Xie et al 2013	Nanxiong	Non-accidental	both	75+	1.09 (0.41, 2.92)	cumulative	27	1.012 (0.986, 1.039)
Xie et al 2013	Taishan	Non-accidental	both	65–74	0.90 (0.40, 2.01)	cumulative	27	↴
Xie et al. 2013	Taishan	Non-accidental	both	75+	2.42 (1.50, 3.90)	cumulative	27	1.017 (0.981, 1.054)
Ma et al. 2012	Shanghai	Non-accidental	both	65+	1.14 (1.08, 1.21)	daily	0	1.140 (1.074, 1.210)
Revich and Shaposhnikov 2010	East Siberia	Non-accidental	both	65+	1.33 (1.06, 1.60)	daily	0	1.330 (1.060, 1.600)
Borst et al 1997	Netherlands	All	both	65–74	1.11 (0.98, 1.27)	daily	0	↴
Borst et al. 1997	Netherlands	All	both	75–84	1.26 (1.18, 1.35)	daily	0	↴
Borst et al. 1997	Netherlands	All	both	85–94	1.25 (1.17, 1.33)	daily	0	↴
Borst et al. 1997	Netherlands	All	both	95+	1.44 (1.23, 1.68)	daily	0	1.937 (1.352, 2.775)
Revich and Shaposhnikov 2008	Moscow	Non-accidental	both	75+	1.099 (1.080, 1.118)	cumulative	11	1.009 (1.007, 1.010)
Revich and Shaposhnikov 2008	Moscow	Non-accidental	both	75+	1.089 (1.067, 1.110)	cumulative	15	1.006 (1.004, 1.007)
Huynen et al. 2001	Netherlands	All	both	65+	1.116 (0.921, 1.505)	daily	0	1.116 (0.827, 1.505)

Abbreviations: Rate Ratio, mortality-rate ratio, derived from the original article; Summary-Rate Ratio, study-specific effect estimate used in the meta-analysis; 95% CI, 95% confidence interval; "↴" indicates that more than one value contributed to the study-specific effect estimate presented below the arrow. Definitions: Effect Type = cumulative if the effect estimate presented in the study was calculated for a time period; Effect Type = daily if the effect was estimated per one day; Lag Days, the number of days used in the conversion of a cumulative measure to a daily measure.

Table S8. Characteristics of studies included in the systematic review but not in a group, complementary information.

Source	Location	Time period	Definition of cold spell (n of episodes)	Main outcomes and stratification by gender and age	Potential confounders taken into account	Main findings and effect modification
Díaz et al. 2006	City of Madrid, Spain	1986-1997	"Cold Wave": daily T_{max} < 6°C; Duration: not defined a priori; (n= not available)	Mortality: All non-accidental, CVD; Male and female	NO_X, SO_2, O_3, TSP, RH, Temporality, Influenza	Female > male

Complementary information related to mortality or morbidity caused by cold spells. Diaz et al. (2006) does not fulfil the criteria for any of the main groups (Overall-effect Group, Added-effect Group, Temperature-change-effect Group). Abbreviations: T_{max}, daily maximum temperature; CVD, cardiovascular diseases; NO_X, nitrogen oxides; SO_2, sulfur dioxide; O_3 ozone, TSP, total suspended particulate matter; Temporality, long- or short term temporal trends and/or seasonal variation and/or day of the week; Influenza, the days or cases associated with influenza epidemics.

Table S9. Estimation of mortality in the index periods (cold spells) and reference periods for the 9 studies in the meta-analyses.

Source	Cold spell: Type	Cold spell: Mortality	Reference period: Type	Reference period: Mortality
Xie et al. 2013	Using frequency distribution of all days of the study period statistically identifies winter cold spells; rarity is influenced by the duration of the study period and by the minimum duration of cold spell	Observed M during ≥ 5 days with daily T_{min} < 5th %centile	Annual cycle-based, Counterfactual inference	Expected M calculated using a 31-day moving average of daily M for cold-spell days of the 2 preceding years and 1 subsequent year combined
Ma et al. 2012	Using frequency distribution of all days of the study period statistically identifies winter cold spells; rarity is influenced by the duration of the study period and by the minimum duration of cold spell	Observed M during ≥ 7 days with daily T_{ave} < 3rd %centile	Seasonally and weekly standardized, Counterfactual inference	Observed M of near-term period with same duration and DOW than cold spell, preceding and following the cold spell, same year. Sensitivity analyses were conducted using same calendar days as the cold spell for previous years
Revich and Shaposhnikov 2010	Using historic frequency distribution statistically identifies rare winter cold spells	Observed M during ≥ 9 days with daily T_{mean} < 3rd %centile, of which ≥ 3 d with daily T_{mean} < 1st %centile	Annual cycle-based, Counterfactual inference	Expected M calculated using a deseasonalizing smooth function (df=128 half a year); a proxy for expected background mortality during cold spell episodes
Chen et al. 2010	Identification does not necessarily depend on the season or the duration of the study period	Observed M 14 days after a temperature drop of >8°C or 14 days after T_{min} <10°C	Seasonally and annually standardized, Counterfactual inference	Observed M 14 days preceding the event
Yang et al. 2009	Identification does not necessarily depend on the season or the duration of the study period	Observed M 14 days after a temperature drop of >8°C or 14 days after T_{min} <10°C	Seasonally and annually standardized, Counterfactual inference	Observed M 14 days preceding the event
Kyselý et al. 2009	Applying absolute T threshold to all study days identifies winter cold spells; rarity does not depend on the duration of the study period	Observed M during ≥ 3 days with daily T_{max} < -3.5°C	Annual cycle-based, Counterfactual inference	Expected M calculated using a deseasonalizing smooth function; a proxy for expected background mortality for each cold spell day
Revich and Shaposhnikov 2008	Using historical frequency distribution of each month statistically identifies rare cold spells for corresponding month during study period	Observed M during ≥ 9 days with daily T_{ave} < 3rd percentile of which ≥ 6 d with daily T_{ave} < 1st %centile	Annual cycle-based, Counterfactual inference	Expected M calculated using same calendar days during the other years of the study period; a proxy for expected background mortality
Huynen et al. 2001	Applying absolute T threshold to all study days identifies winter cold spells; rarity does not depend on the duration of the study period	Observed M during ≥ 9 days with daily T_{min} ≤ -5°C, of which ≥ 6 days with daily T_{min} ≤ -10°C	Annual cycle-based, Counterfactual inference	Expected M calculated using a 31-day moving average of daily M for cold spell days of the 2 preceding years combined
Borst et al. 1997	A proxy for winter cold spells; rarity does not depend on the duration of the study period or the duration of the cold spell	Observed M during all 7 day-periods with average of weekly T_{max} < 5°C	Period of lowest mortality, Counterfactual inference	Observed M during all 7 day-periods with average of weekly T_{max} 15.0-19.9C; optimum temperature range defined by lowest mortality in 6 temperature strata

Abbreviations: M, mortality; T_{min}, daily minimum temperature; T_{ave}, daily average temperature; DOW, day of the week; T_{mean}, daily mean temperature; df, degrees of freedom; T_{max}, daily maximum temperature. Definitions: Annual cycle-based, selection of the reference period takes into account potential annual cycles by using the same calendar days as the exposure from other years; Seasonally standardized, selection of the reference period takes into account potential seasonal variation by using near-term days of the same year or other years; Annually standardized, selection of the reference period takes into account potential differences between years by using days of the same year as the exposure; Weekly standardized, selection of the reference period takes into account potential variation by weekday by using same days of the week as the exposure, for the same year or other years; Rarity depends on the duration of the study period, the absolute number of days below a statistically defined percentile becomes greater if the number of days in the frequency distribution is increased. Rarity is still influenced by definition of minimum duration of the event, as it can't be controlled which portion of the individual cold days occur consecutively and which separately.

References

Borst V, Schols JMGA, Mackenbach JP. 1997. Increased mortality among nursing home patients during ambient temperature extremes; more rise during hot than during cold spells. Ned Tijdschr Geneeskd 141:2180–2183.

Chen VY, Wu PC, Yang TC, Su HJ. 2010. Examining non-stationary effects of social determinants on cardiovascular mortality after cold surges in Taiwan. Sci Total Environ 408:2042–2049.

Díaz J, Linares C, Tobías A. 2006. Impact of extreme temperatures on daily mortality in Madrid (Spain) among the 45-64 age-group. Int J Biometeorol 50:342–348.

Huynen MM, Martens P, Schram D, Weijenberg MP, Kunst AE. 2001. The impact of heat waves and cold spells on mortality rates in the Dutch population. Environ Health Perspect 109:463–470.

Kyselý J, Pokorna L, Kyncl J, Kriz B. 2009. Excess cardiovascular mortality associated with cold spells in the Czech Republic. BMC Public Health 9:19.

Ma W, Yang C, Chu C, Li T, Tan J, Kan H. 2012. The impact of the 2008 cold spell on mortality in Shanghai, China. Int J Biometeorol 57:179–84. doi:10.1007/s00484-012-0545-7.

Revich B, Shaposhnikov D. 2008. Excess mortality during heat waves and cold spells in Moscow, Russia. Occup Environ Med 65:691–696.

Revich BA, Shaposhnikov DA. 2010. Extreme temperature episodes and mortality in Yakutsk, East Siberia. Rural Remote Health [internet] 10:1338. Available: http://www.rrh.org.au/articles/subviewnew.asp?ArticleID=1338.

Xie H, Yao Z, Zhang Y, Xu Y, Xu X, Liu T, et al. 2013. Short-term effects of the 2008 cold spell on mortality in three subtropical cities in Guangdong Province, China. Environ Health Perspect 121:210–216.

Yang TC, Wu PC, Chen VY, Su HJ. 2009. Cold surge: a sudden and spatially varying threat to health? Sci Total Environ 407:3421–3424.
